# Clonal relatedness of coagulase-positive staphylococci among healthy dogs and dog-owners in Spain. Detection of multidrug-resistant-MSSA-CC398 and novel linezolid-resistant-MRSA-CC5

**DOI:** 10.3389/fmicb.2023.1121564

**Published:** 2023-03-02

**Authors:** Idris Nasir Abdullahi, Carmen Lozano, Myriam Zarazaga, Andre Becker Simoes Saidenberg, Marc Stegger, Carmen Torres

**Affiliations:** ^1^Area of Biochemistry and Molecular Biology, OneHealth-UR Research Group, University of La Rioja, Logroño, Spain; ^2^Department of Bacteria, Parasites, and Fungi, Statens Serum Institut, Copenhagen, Denmark; ^3^Section for Food Safety and Zoonoses, Institute for Veterinary and Companion Animal Science, Københavns Universitet, Copenhagen, Denmark

**Keywords:** *Staphylococcus*, zoonosis, pets, MSSA-CC398, *Staphylococcus pseudintermedius* carriage, *Staphylococcus aureus* carriage, linezolid resistance

## Abstract

**Introduction:**

Nasal carriage of coagulase-positive staphylococci (CoPS) in healthy dogs could indicate increased risks of colonization for in-contact people or vice versa. This study determined the nasal carriage rate of CoPS among healthy dogs and in-contact people, their genotypic characteristics and phylogenetic relatedness.

**Methods:**

Nasal samples were collected from 27 households (34 dogs and 41 humans) in Spain. Staphylococci were identified by MALDI-TOF-MS, their antimicrobial resistance (AMR) genes and *spa*-types were tested by PCR/sequencing. The relatedness of CoPS from the same households was assessed by core genome single nucleotide polymorphisms (SNPs) analyses.

**Results:**

*Staphylococcus aureus* carriage was found in 34.1% of humans (including one methicillin-resistant *S. aureus* MRSA-CC5-t2220-SCC*mec* type-IV2B) and 5.9% of dogs; *Staphylococcus pseudintermedius* in 2.4% of humans and 32.4% of dogs; while *Staphylococcus coagulans* was only detected in dogs (5.4%). Remarkably, one human co-carried *S. aureus*/*S. pseudintermedius*, while a dog co-carried the three CoPS species. Household density was significantly associated with *S. pseudintermedius* carriage in households with > than 1 dog and >than 1 human (OR = 18.10, 95% CI: 1.24–260.93, *p* = 0.034). Closely related (<15 SNPs) *S. aureus* or *S. pseudintermedius* were found in humans or dogs in three households. About 56.3% *S. aureus* carriers (dog or human) harboured diverse within-host *spa*-types or AMR genotypes. Ten clonal complexes (CCs) were detected among the *S. aureus*, of which methicillin-susceptible *S. aureus*-CC398-IEC-type C (t1451 and t571) was the most frequent, but exclusive to humans. *S. aureus* and *S. pseudintermedius* isolates harboured resistance genes or mutations associated to 9 classes of antimicrobials including linezolid (G2261A & T1584A point mutations in 23S rDNA). The *S. coagulans* isolates were susceptible to all antimicrobials. Most of the *S. pseudintermedius* carried *lukS/F-I, siet,* and *sient* genes, and all *S. aureus* were negative for *lukS/F*-PV*, tst-1, eta* and *etb* genes.

**Discussion:**

Clonally related human-to-human MSSA and dog-to-human MSSP were found. The detection of the MSSA-CC398 clade highlights the need for its continuous surveillance from One Health perspective.

## Introduction

Resident bacteria of the mucosal surfaces (such as nasal or oral) and skin of dogs could easily be transmitted to in-contact persons or owners by direct contact or from the household environment (Trinh et al., 2018). Moreover, transmission from humans to pets could also be possible (Orsini et al., 2022). These events (zoonosis and anthroponosis) represent a growing public health problem (Rahman et al., 2020; Orsini et al., 2022).

Two main groups of staphylococci are defined based on rabbit’s plasma coagulase activity, *viz*, coagulase-negative staphylococci (CoNS) and coagulase-positive staphylococci (CoPS) (Carroll et al., 2021). Of the two groups, CoPS are more pathogenic (Carroll et al., 2021). *Staphylococcus aureus* is the archetype of the CoPS and relevant animal and human pathogen (Tong et al., 2015); however, other CoPS related to animals have been described. One of the emerging CoPS of clinical importance is *S. pseudintermedius*, a member of the *Staphylococcus intermedius* group (SIG) that comprises four other species, *viz.*; *Staphylococcus intermedius, Staphylococcus delphini,* the recently described *Staphylococcus cornubiensis*, and *S. ursi* (Murray et al., 2018; Perreten et al., 2020; Carroll et al., 2021). In addition to these CoPS, *Staphylococcus coagulans* is another species that was originally described in 1990 as *Staphylococcus schleiferi* subsp. *coagulans* before being classified, based on genomic studies, into a separate species in 2020 (Madhaiyan et al., 2020).

*Staphylococcus aureus* is an opportunist pathogen frequently found in humans, which colonizes the anterior nose and nasal vestibule of 25–50% of healthy people (Liu et al., 2015; Sakr et al., 2018). *S. aureus* nasal colonization in humans and animals could place individuals at risk of opportunistic infections, and the carriage of specific virulence genes by the isolates can increase their pathogenic potential. In this respect, the following virulence genes are especially relevent in *S. aureus*: *tst* (encodes the toxic shock syndrome toxin, associated with sepsis), *eta* and *etb* (encode the exfoliatin A and B, associated with the staphylococcal scalded skin syndrome), and the *luk-S/F-PV* (encodes Panton Valentine Leucocidin, associated with abscesses and community-acquired pneumonia) (Tam and Torres, 2019; Hu et al., 2021).

The population genetics of *S. aureus* have shown the presence of several clonal complexes (CCs). Remarkably, some of these CCs have somewhat animal host specificity, while others are less host-specific (Chaguza et al., 2022). Methicillin-Resistant *S. aureus* (MRSA) is a globally epidemic bacterium in nosocomial settings and is referred to as healthcare-associated MRSA (HA-MRSA) (Cuny et al., 2010; Cuny et al., 2022). MRSA has also emerged in the community without any relation to the healthcare facilities [community associated (CA)-MRSA] and in the last decade, some CCs (mainly CC398) have been associated with livestock animals (LA-MRSA) (Matuszewska et al., 2022).

*Staphylococcus aureus* frequently colonizes the skin and nasal cavity of healthy humans, while *S. pseudintermedius* appears to predominantly colonize pets (especially dogs) but could occasionally be found in humans (Cuny et al., 2022). Although infrequent, *S. pseudintermedius* has been also isolated from human skin lesions (Lozano et al., 2017), due en ocassions to dog bites (Börjesson et al., 2015), as well as causing septicemia (Somayaji et al., 2016). Previous reports on nasal colonization in humans by *S. pseudintermedius* in dog-owning households have reported a prevalence of 25–65.9% in dogs and 3–4.5% in humans (Gómez-Sanz et al., 2013c; Han et al., 2016; Rodrigues et al., 2018). Certain isolates of *S. pseudintermedius* have become a major veterinary pathogen of concern due to their frequent multidrug-resistance phenotypes (Priyantha, 2022). Also, severe human infections with methicillin-resistant *S. pseudintermedius* (MRSP) have recently been reported (Priyantha, 2022).

Data about the co-colonization of dogs and in-contact persons with CoPS at the community level are still scarce. Hanselman et al. (2009) reported the presence of *S. pseudintermedius*, *S. aureus*, and S. *schleiferi* subsp. *coagulans* (including methicillin-resistant isolates) in dogs at the household level. In addition, concurrent animal and human colonization by indistinguishable *S. pseudintermedius* and *S. aureus* isolates have been observed (Hanselman et al., 2009). In a previous study performed a decade ago, our research group investigated the household nasal carriage of CoPS in dogs and humans, determining the prevalence of genetic lineages and their recovery rate over a year in animals and owners (Gómez-Sanz et al., 2013c). Nowadays, the abundance of dog ownership in Spain has significantly changed and the number of households with pets has increased. According to available data, there were 20 million registered pets in Spain in 2019 and about 26% of all households own dogs (Asociación Madrileña de Veterinarios de Animales de Compañía, 2019). By implication, sharing and transmission of nasal microorganisms (such as CoPS) between dogs and dog-owning households could be more frequent (Van Balen et al., 2017).

Nasal carriage of CoPS in healthy dogs could be an indicator of increased risk of colonization/infection for people in-contact with these animals, especially if *S. aureus* carries key virulence determinants of special relevance such as *lukS/F*-PV, *tst-1, eta* and *etb* (González-Martín et al., 2020). However, the influence of pet ownership on the diversity of CoPS isolates circulating among dog owners still needs to be fully elucidated (Van Balen et al., 2017). Consequently, this study sought to understand the current epidemiological situation of CoPS nasal carriage in dog-owning households by characterization of the genetic lineages, relatedness between isolates from the same households, antimicrobial resistance (AMR) determinants, virulence factors, and immune evasion cluster (IEC) types of *S. aureus* isolates.

## Materials and methods

### Samples analyzed and staphylococci recovery

A total of 41 humans and 34 dogs from 27 households were prospectively studied to determine the nasal carriage of CoPS; the sampling was performed in La Rioja region (Northern Spain) between January to March 2022. Household density was classified into four, *viz*: (a) household with a dog and a human (b) household with >1 dog and a human, (c) household with 1 dog and >than 1 human, and (d) household with > than 1 dog and >than 1 human. The sampled humans and dogs did not have recent hospital stays prior to the study or received antibiotics (at least 3 months before sampling) and the humans had no professional contact with health institutions and did not work in microbiology laboratories. None of the participants had consultations or visits to hospitals in the last 3 months before sample collection. Nasal samples were obtained using sterile swabs with conservation media (Amies BD Life sciences®, New Jersey, USA). All procedures were approved by the ethical committee of the University of La Rioja (Spain) and were carried out following all applicable international, national, and/or institutional guidelines for human samples experiments (as described in the revised Helsinki declaration) and for ethical use of animals (directive 2010/63/EU, Spanish laws 9/2003 and 32/2007, and RD 178/2004 and RD 1201/2005).

Samples were enriched in Brain Heart Infusion broth (BHI; Condalab, Madrid, Spain) supplemented with 6.5% (w/v) NaCl and incubated for 24 h at 37°C. After 24 h of incubation, the broth samples were diluted and carefully dispensed onto four different bacteriological media: blood agar, mannitol salt agar (MSA; Condalab, Madrid, Spain), oxacillin resistance screening agar base (ORSAB with 2 mg/L oxacillin; Oxoid, Hampshire, UK) and ChromAgar LIN (Paris, France) and incubated for 24 h at 37°C. After overnight growth, 3 to 8 different colonies (presenting staphylococci morphology) were randomly selected per sample and identified by matrix-assisted laser desorption/ionization time-of-flight mass spectrometry (*MALDI-TOF,* Bruker Daltonics, Bremen, Germany) using the standard extraction protocol. Those isolates identified as CoPS were included in this study.

### Antimicrobial susceptibility testing

Susceptibility testing for 13 antimicrobial agents was performed by the disk diffusion method following the recommendations and breakpoints of the European Committee on Antimicrobial Susceptibility Testing (European Committee on Antimicrobial Susceptibility Testing (EUCAST), 2022). The antimicrobial agents tested were as follows (μg/disk): penicillin (PEN) (1 or 10 μg, depending on the CoPS species), cefoxitin (FOX) (30 μg to detect methicillin-resistant *S. aureus* (MRSA) isolates), oxacillin (OXA) (1 μg to detect methicillin-resistant *S. pseudintermedius* [MRSP] or *S. coagulans* isolates), erythromycin (ERY) (15 μg), clindamycin (CLI) (2 μg), gentamicin (GEN) (10 μg), tobramycin (TOB) (10 μg), tetracycline (TET) (30 μg), ciprofloxacin (CIP) (5 μg), chloramphenicol (CHL) (30 μg), linezolid (LZD) (10 μg), mupirocin (MUP) (200 μg), and trimethoprim-sulfamethoxazole (SXT) (1.25 μg + 23.75 μg).

Once the antimicrobial resistance phenotype of all CoPS was determined, distinct isolates were selected for further studies (defined as those of different samples or those from the same sample but of different species, and/or different antimicrobial resistance phenotypes).

### DNA extraction (for PCR)

For DNA extraction, isolates were seeded on BHI agar and incubated for 24 h at 37°C. An isolated colony was suspended in 45 μL of sterile MiliQ water and added 5 μL of lysostaphin (1 mg/mL) (Sigma). The mixture was vortexed and incubated for 10 min at 37°C. Forty-five μL of sterile MiliQ water, 150 μL of Tris–HCl (0.1 M, pH 8) and 5 μL of proteinase K (2 mg/mL) (Sigma) were added. The final mixture was vortexed and incubated for 10 min at 60°C, then boiled for 5 min at 100°C. To separate and obtain the DNA (supernatant) from debris, the final mixture was centrifuged at 12,000 revolutions per minute for 3 min, and stored at −20°C.

### Study of antimicrobial resistance genes

The presence of the following resistance genes was tested by single PCRs, selected according to the antimicrobial resistance phenotype of isolates: beta-lactams (*blaZ, mecA,* and *mecC*), erythromycin and clindamycin (*ermA, ermB, ermC, ermT, mphC, msrA, lnuA,* and *lnuB*), aminoglycosides (*aac6′-aph2″,* and *ant4′*), tetracycline [*tet*(L)*, tet*(M)*,* and *tet*(K)], trimethoprim (*dfrA, dfrD, dfrG* and *dfrK*), and chloramphenicol (*catpC221, catpC223, catpC194, catA, fexA,* and *fexB*). Linezolid or chloramphenicol-resistant isolates were screened for the presence of the linezolid transferable resistance genes (*cfr, cfrB, cfrD, poxtA,* and *optrA*). Mutations in 23S rDNA were also investigated by PCR and amplicon sequencing. The obtained sequences were compared with those of linezolid-susceptible *S. aureus* NCTC 8325 (GenBank accession number CP000253) using the EMBOSS Needle software for nucleotide and amino acid (BLOSUM62 cost matrix) alignments. Primers and conditions of PCRs performed in this study are included in Supplementary Table S1. Isolates were considered multi-drug resistant (MDR) when they were resistant to ≥3 classes of the antimicrobial agents tested (Magiorakos et al., 2012).

### Detection of virulence and toxin genes of CoPS

The presence of the genes *tst* (toxin of shock toxic syndrome)*, lukS/F*-PV (Panton-Valentine leucocidin), and *eta* and *etb* (exfoliative toxins A and B), was tested by PCR. Immune evasion cluster (IEC) genes (*scn, chp, sak, sea,* and *sep*) were analysed and classified into seven different IEC types (A–G), based on the combination of the positive genes. The *scn* gene (encoding the staphylococcal complement inhibitor) was used as a marker of the IEC system (van Wamel et al., 2006). The presence of the *lukS/F-I, siet,* and *sient* genes was analysed for the *S. pseudintermedius* isolates. Primers and conditions of PCRs performed in this study are included in Supplementary Table S1.

### Molecular typing of isolates

All recovered *S. aureus* isolates were characterized by *spa* typing by the PCR/Sanger sequencing. New repeat combinations were submitted to the Ridom *spa* Server.^1^ CC398 clone was determined by a specific PCR protocol for the *sau1*-*hsdS1* variant (Stegger et al., 2011). The clonal complex of the isolates was assigned, when possible, according to the *spa*-types. Moreover, multilocus sequence typing (MLST) was performed in selected *S. aureus* isolates (isolates with *spa*-types repeatedly found and those with new *spa-*types). The seven housekeeping genes of *S. pseudintermedius* (*pta, cpn60, tuf, ack, purA, sar* and *fdh*) were amplified, and the sequence type (ST) was assigned according to the MLST database.^2^ The selection of *S. pseudintermedius* isolates for MLST was only on household with dog and human carriers. Staphylococcal Cassette Chromosome *mec* (SCC*mec*) types were determined by multiplex PCRs. Primers and conditions of PCRs performed in this study are included in Supplementary Table S1.

Positive controls (confirmed by sequencing) from the collection of the Universidad de La Rioja were included in all the PCR assays in this study.

### Whole genome sequencing

Fourteen selected isolates (11 *S. aureus* and two *S. pseudintermedius*) were whole genome sequenced on the Illumina NextSeq platform. The selection was based on: (a) all MSSA-CC398 isolates, (b) all isolates from households with two or more humans and/or dogs’ carriers of either *S. aureus* or *S. pseudintermedius*, (c) the MRSA isolate.

The MagNA Pure 96 DNA Multi-Sample Kit (Life Technologies, Carlsbad, CA, USA, 4413021) was used to extract genomic DNA according to instructions provided by the manufacturers. The Qubit 1X dsDNA HS Assay Kit (Thermo Fisher Scientific, Scoresby, VIC, Australia) was used for DNA quantification, while Sequencing libraries were prepared using the Illumina Nextera XT DNA Library Preparation Kit (Illumina, San Diego, CA, USA, FC-131-1096) and sequenced on the NextSeq 500 platform (Illumina, San Diego, CA, USA) using a 300-cycle kit to obtained paired-end 150 bp reads, as previously described (Saidenberg et al., 2022).

### Genomic assembly and phylogenetic analysis

All the genomes analysed in this study were *de novo* assembled using SPAdes (v.3.15.5), performing the *in silico* typing with the settings of a minimum of 90% coverage and 80% identity. Core-genome single nucleotide polymorphisms (SNPs) were detected with the NASP pipeline v.1.0.0 (Sahl et al., 2016), mapping against the chromosome reference sequences CA-347 (GenBank accession ID CP006044, *S. aureus*), and SP_11304-3A (GenBank accession ID CP065921.1, *S. pseudintermedius*) for each separate phylogenetic analysis. GATK (v.4.2.2) was used to call SNPs and excluded positions featuring <90% unambiguous variant calls and <10 depth. IQ-TREE (v.2.1.2), was used to construct the phylogenetic trees using ModelFinder with 100 bootstraps. The graphical data was added to the phylogenies with iTOL v.6.6 (Letunic and Bork, 2021).^3^

### Genomic typing

The STs were determined with MLST v.2.16 (Jolley et al., 2018),^4^ and undefined STs were submitted to the PubMLST database for ST assignment (Larsen et al., 2012). Virulence factors, plasmid replicons, and antimicrobial resistance genes were identified using ABRicate v.0.9.0 and the respective databases VFDB, Plasmidfinder, and Resfinder databases from the Center for Genomic Epidemiology.^5^^,^^6^ The *spa*Typer v1.0 was used to confirm the *spa* types (Bartels et al., 2014) and mutations associated with AMR were identified using ResFinder v4.1 (Bortolaia et al., 2020) and PointFinder (Zankari et al., 2017).

### Genome availability

All the raw genome reads generated from this study have been deposited at European Nucleotide Archive under Study Accession no. PRJEB57210.^7^

### Data management and statistics

Data collected were verified and processed and the Statistical Package for Social Sciences (SPSS) Version 26 (IBM, California, U.S.A) was used for analysis. Data were reported as numbers and percentages (for categorical variables) and presented on tables and charts. Data were subjected to univariate logistic regression to compute odd ratio (OR) at a 95% confidence interval (95%CI) between the carriage rate of *S. aureus*/*S. pseudintermedius* and the household densities with significant association (*p* < 0.05).

## Results

### CoPS nasal carriage in healthy dogs’ households

A total of 73 *S. aureus,* 31 *S. pseudintermedius* and two *S. coagulans* isolates were recovered from 75 nasal samples of humans and dogs. After AMR phenotype determination, 52 isolates were selected for further characterization (31 *S. aureus*, 19 *S. pseudintermedius* and 2 *S. coagulans*), corresponding to one isolate per sample or more than one if they presented different species and/or different AMR phenotype (Supplementary Table S2).

**Figure 1:**
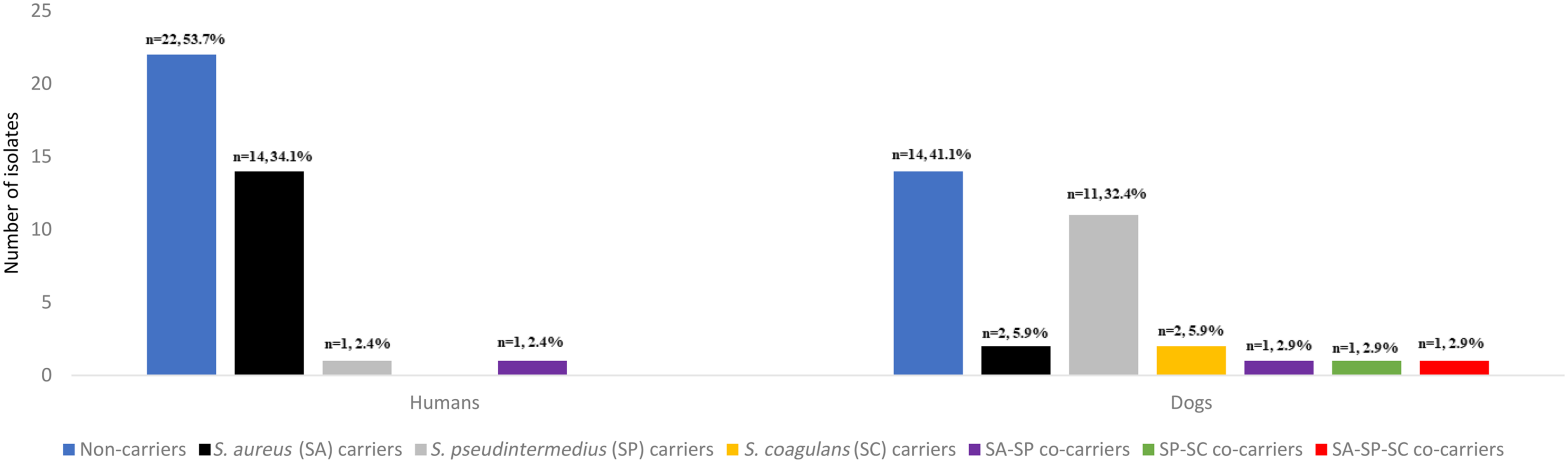
Number and prevalence of *Staphylococcus aureus*, *Staphylococcus pseudintermedius*, *Staphylococcus coagulans* isolates and co-carriage detected in the nasal cavities of healthy humans and dogs.

*Staphylococcus aureus* was found in 14 humans (34.1%) (including one individual with MRSA) and two dogs (5.9%) (Figure 1). *S. pseudintermedius* was identified in one human (2.4%) and 11 dogs (32.4%). However, *S. coagulans* was solely identified in two dogs (5.9%) of the same household. Apart from these three species, no other CoPS species were detected in the cultures. Remarkably, one human presented *S. aureus*/*S. pseudintermedius* co-carriage (2.4%) while a dog had co-carriage of all three CoPS species (2.9%) (Figure 1). In total, 14 humans and 12 dogs carried CoPS. Household density was significantly associated with *S. pseudintermedius* carriage in households with > than 1 dog and >than 1 human (OR = 18.10, 95% CI: 1.24–260.93, *p* = 0.034) (Table 1).

**Table 1:** Association of household density with nasal *S. aureus* and *S. pseudintermedius* carriage.

Household density	*S. aureus* carriers. Number (%)	*S. aureus* non-carriers. Number (%)	OR (95% CI)	*p* value	*S. pseudintermedius* carriers. Number (%)	*S. pseudintermedius* carriers. Number (%)	OR (95% CI)	*p* value
1 dog and 1 human (*n* = 10)	3 (30)	7 (70)	Referent	Referent	1 (10)	9 (90)	Referent	Referent
>1 dog and 1 human (*n* = 3)	3 (100)	0 (0)	15.0 (0.59–376.7)	0.099	1 (33.3)	2 (66.7)	4.5 (0.19–106.8)	0.352
1 dog and >1 human (*n* = 8)	2 (25)	6 (75)	0.8 (0.09–6.32)	0.814	2 (25)	6 (75)	3.0 (0.22–40.93)	0.410
>1 dog and >1 human (*n* = 6)	5 (83.3)	1 (16.7)	11.7 (0.92–147.57)	0.057	4 (66.7)	2 (33.3)	18.0 (1.24–260.93)	0.034*

*Significant association determined by bivariate regression at 95% confidence interval (CI).

### Phenotypic and genetic characteristics of CoPS isolates

**Figure 2:**
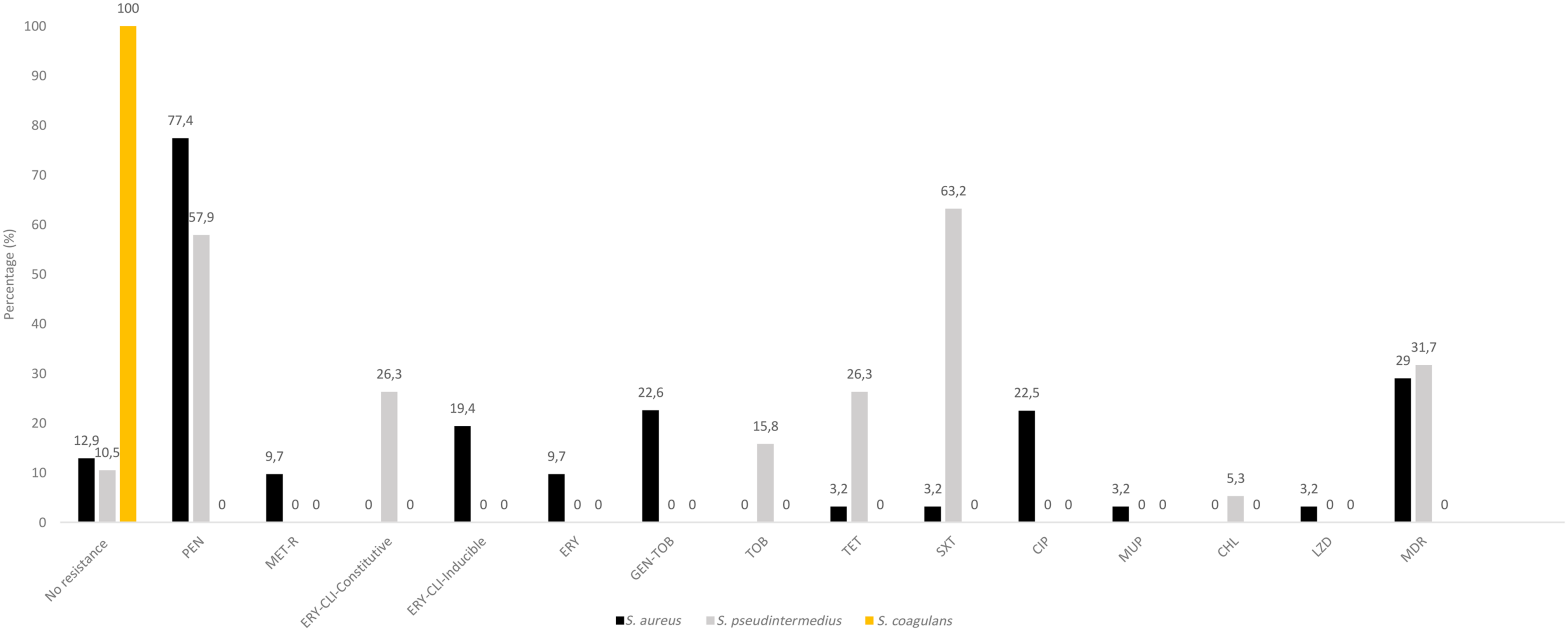
Antimicrobial resistance rates in *S. aureus*, *S. pseudintermedius* and *S. coagulans* isolates. Percentages were based on the collection of CoPS (31 *S. aureus*, 19 *S. pseudintermedius* and 2 *S. coagulans*) obtained of different samples or those of the same sample but with different species and/or AMR phenotype. CHL, chloramphenicol; CLI, clindamycin; CIP, ciprofloxacin; ERY, erythromycin; GEN, gentamicin; MET-R, methicillin-resistant; MDR, multidrug resistance (resistance to three or more classes of antibiotics); PEN, penicillin; SXT, sulfamethoxazole/trimethoprim; TET, tetracycline, TOB, tobramycin.

The 31 distinct *S. aureus* isolates harboured AMR as follows [percentage of resistant isolates/resistance genotype]: penicillin [77.4/*blaZ*], cefoxitin [9.7/*mecA*], erythromycin-clindamycin-inducible [19.4/*ermT*], erythromycin [9.7/*msrA, mphC*], clindamycin [3.2/*lnuA*], gentamicin-tobramycin [22.6/*aac6′-aph2″*], tetracycline [3.2/*tet*(K)], sulfonamide [3.2/*dfrA*], fluoroquinolones [22.5/amino acid changes in GrlA: S80F, GyrA: S84L], mupirocin (3.2/*mupA*) and linezolid [3.2/G2261A & T1584A point mutations in 23S rDNA] (Figure 2; Table 2). Moreover, the 19 distinct *S. pseudintermedius* isolates harboured AMR as follows [percentage of resistant isolates/resistance genotype]: penicillin [57.9/*blaZ*], erythromycin-clindamycin-constitutive [26.3/*ermB*], tobramycin [15.8/*ant4′*], tetracycline [26.3/*tet*(M)], trimethoprim-sulfamethoxazole [63.2/*dfrA, dfrD, dfrG, dfrK*], and chloramphenicol [5.3/*catA*] (Figure 2; Table 3). No resistance markers were detected in the *S. coagulans* isolates, that were susceptible to all antimicrobial agents tested (Figure 2).

**Table 2:** Molecular characterization, antimicrobial resistance and virulence profile of the *S. aureus* isolates from humans and dogs.

Host/ID No.	Household ID N^o^/population in household	N^o^ of isolates	*spa* type	CC	AMR phenotypes	Methicillin susceptibility	MDR phenotype	AMR genes detected	IEC genes/type	Virulence genes detected (*lukS/F*-*PV, tst, eta, etb*)
Human/3	2/1 human and 1 dog	3	t2220	CC5	PEN^3^-FOX^3^-ERY^3^-CIP^3^-GEN^2^-TOB^2^-MUP^1−^LZD^1^	MRSA-SCC*mec* type-IV (2B)	Yes	*blaZ*^3^*, mecA*^3^*, msrA^3^, mphC*^3^ GrlA (S80F)^1^, GrlA (S84L)^1^*, aac6′-aph2″*^2^*, mupA*^1^, G2261A & T1584A point mutations in 23SrDNA^1^	Negative	Negative
		1	t012	CC30	PEN	MSSA	No	*blaZ*	Negative	Negative
Human/9	5/1 human and 2 dogs	1	t012	CC30	PEN-CIP-TET	MSSA	Yes	*blaZ, tet*(K)	Negative	Negative
		1	t012	CC30	PEN-CIP	MSSA	No	*blaZ*	Negative	Negative
		1	t1824	CC30	PEN	MSSA	No	*blaZ*	*scn, sak, sep*/G	Negative
Human/17	8/2 humans and 2 dogs	1	t4735	CC133	Susceptible	MSSA	No	NT	Negative	Negative
		1	t4735	CC133	CIP-SXT	MSSA	No	*dfrA*	Negative	Negative
Human/20	9/1 human and1 dog	1	t3092	CC8	PEN	MSSA	No	*blaZ*	Negative	Negative
		1	t068	CC8	PEN	MSSA	No	*blaZ*	*scn, sak, sep*/G	Negative
Human/23	10/2 humans and 2 dogs	1	t267	CC97	Susceptible	MSSA	No	NT	*scn, sak*/E	Negative
		1	t267	CC97	Susceptible	MSSA	No	NT	*scn, sak*/E	Negative
Dog/24		1	t2013	CC15	PEN	MSSA	No	*blaZ*	*scn, chp*/C	Negative
Human/26	11/2 humans and 2 dogs	1	t1070	CC30	PEN	MSSA	No	*blaZ*	Negative	Negative
Human/27		1	t1070	CC30	PEN	MSSA	No	*blaZ*	Negative	Negative
Dog/29		1	t121	CC8	PEN-CIP	MSSA	No	*blaZ*, GrlA (S80F)	*scn, sak, sea*/D	Negative
Human/30	12/1 human and 1 dog	1	t041	CC5	Susceptible	MSSA	No	NT	*scn, sak*/E	Negative
Human/38	15/1 human and 2 dogs	1	t505	CC45	PEN	MSSA	No	*blaZ*	*scn, sak*/E	Negative
		1	t065	CC45	PEN	MSSA	No	*blaZ*	*scn, sak*/E	Negative
		1	t015	CC45	PEN	MSSA	No	*blaZ*	*scn, sak, sep*/G	Negative
Human/44	17/1 human and 1 dog	1	t571	CC398	ERY-CLI^ind^	MSSA	No	*ermT*	*scn, chp*/C	Negative
Human/50	19/2 humans and 2 dogs	1	t1689	CC45	PEN	MSSA	No	*blaZ*	*scn, sak*/E	Negative
		1	t091	CC7	PEN	MSSA	No	*blaZ*	*scn, sak, sep*/G	Negative
Human/57	21/2 humans and 1 dog	1	t1451	CC398	PEN-ERY-CLI^ind^-GEN-TOB	MSSA	Yes	*blaZ, ermT, aac6′-aph2″*	*scn, chp*/C	Negative
		1	t1451	CC398	PEN-ERY-CLI^ind^-GEN-TOB	MSSA	Yes	*ermT, aac6′-aph2″*	*scn, chp*/C	Negative
		1	t571	CC398	PEN-ERY-CLI^ind^-GEN-TOB	MSSA	Yes	*blaZ, ermT, aac6′-aph2″*	*scn, chp*/C	Negative
Human/58		1	t1451	CC398	PEN-ERY-CLI^ind^-GEN-TOB	MSSA	Yes	*blaZ, ermT, aac6′-aph2″*	*scn, chp*/C	Negative
		1	t571	CC398	PEN-ERY-CLI^ind^-GEN-TOB	MSSA	Yes	*blaZ, ermT, aac6′-aph2″*	*scn, chp*/C	Negative
Human/60	22/I human and 1 dog	1	t084	CC15	PEN	MSSA	No	*blaZ*	*scn, chp*/C	Negative
		1	t355	CC152	PEN	MSSA	No	*blaZ*	*scn, sak*/E	Negative

ST, Sequence Type. CHL, chloramphenicol; CLI, clindamycin; CIP, ciprofloxacin; ERY, erythromycin; GEN, gentamicin; PEN, penicillin; SXT, sulfamethoxazole/trimethoprim; TET, tetracycline; TOB, tobramycin; ERY-CLI^ind^, erythromycin-clyndamycin inducible.

**Table 3:** Intra-host variation of AMR determinant and virulence factors of *S. pseudintermedius* isolates.

Host/ID	Household ID N^o^/population	N^o^ of isolates	AMR phenotypes	MDR phenotype	AMR genes detected	Methicillin Susceptibility/ST	Virulence genes detected
Dog/2	1/1 human and 1 dog	4	PEN^4^-ERY^4^-CLI^4^-TET^4^-SXT^1^-TOB^2^	Yes	*blaZ*^4^*, ermB*^4^*, tet*(M)^3^, *ant4′*^2^	MSSP/NT	*lukS/F-I* ^3^ *, siet* ^3^ *, sient* ^4^
Human/23	10/2 humans and 2 dogs	1	Susceptible	No	NT	MSSP/ST1115	*lukS/F-I* ^1^ *, siet* ^1^ *, sient* ^1^
Dog/25	10/2 humans and 2 dogs	1	Susceptible	No	NT	MSSP/ST1115	*lukS/F-I* ^1^ *, siet* ^1^ *, sient* ^1^
Dog/28	11/2 humans and 2 dogs	1	SXT^1^	No	*dfrK* ^1^	MSSP/NT	*lukS/F-I* ^1^*, siet*^1^*, sient*^1^
Dog/29	11/2 humans and 2 dogs	1	SXT^1^	No	ND	MSSP/NT	*lukS/F-I* ^1^*, siet*^1^*, sient*^1^
Dog/43	16/2 humans and 1 dog	1	PEN^1^	No	*blaZ* ^1^	MSSP/NT	*lukS/F-I* ^1^*, siet*^1^*, sient*^1^
Dog/39	15/1 human and 2 dogs	1	PEN^1^-SXT^1^	No	*blaZ* ^1^ *, dfrA* ^1^ *, dfrG* ^1^	MSSP/NT	*lukS/F-I* ^1^*, siet*^1^*, sient*^1^
Dog/39	15/1 human and 2 dogs	1	SXT^1^	No	*dfrA* ^1^ *, dfrG* ^1^	MSSP/NT	*lukS/F-I* ^1^*, siet*^1^*, sient*^1^
Dog/49	18/2 humans and 2 dogs	1	SXT^1^	No	*dfrA* ^1^ *, dfrG* ^1^	MSSP/NT	*lukS/F-I* ^2^*, siet*^2^*, sient*^2^
Dog/48	18/2 humans and 2 dogs	1	TET^1^	No	*tet*(M)*^1^*	MSSP/NT	*lukS/F-I* ^4^*, siet*^4^*, sient^4^*
Dog/52	19/2 humans and 2 dogs	3	PEN^3^-SXT^3^-TOB^1^	Yes	*blaZ* ^3^ *, dfrA* ^1^ *, dfrG* ^1^ *, blaZ^1^, dfrG, ant4′* ^1^	MSSP/NT	*lukS/F-I* ^3^*, siet*^3^*, sient*^3^
Dog/53	19/2 humans and 2 dogs	2	PEN^2^-SXT^2^	No	*blaZ* ^1^ *, dfrG* ^2^ *, dfrK* ^2^	MSSP/NT	*lukS/F-I* ^2^*, siet*^2^*, sient*^2^
Dog/60	22/1 human and 1dog	1	PEN^1^-ERY^1^-CLI^1^-CHL^1^-SXT^1^	Yes	*ermB* ^1^ *, catA* ^1^ *, dfrD* ^1^	MSSP/NT	*lukS/F-I* ^1^*, siet*^1^*, sient*^1^

In superscript is the number of isolates that present the specific phenotype/genotype of AMR. ST, Sequence Type; NT, Not tested; ND, not detected; CHL, chloramphenicol; CLI, clindamycin; ERY, erythromycin; PEN, penicillin; OXA, oxacillin; SXT, sulfamethoxazole/trimethoprim; TET, tetracycline; TOB, tobramycin.

Regarding the genetic lineages of *S. aureus* isolates, the three MRSA isolates from humans (same individual but different AMR phenotypes/genotypes) belonged to the *spa* type t222, associated with CC5. All other isolates were methicillin-susceptible *S. aureus* (MSSA) with 19 different *spa*-types assigned to 10 different CCs. The MSSA-CC398 clone (t1451 and t571) was the most frequently identified (18.8% of *S. aureus* carriers); these isolates were all IEC-type C. Other CCs (*spa*-types) detected were as follows: CC5 (t041), CC7 (t091), CC8 (t121, t126, t1070, t3092), CC15 (t084, t2013), CC30 (t012, t1824), CC45 (t015, t065, t505, t1689), CC97 (t267), CC133 (t4735) and CC152 (t355) (Table 2). For *S. pseudintermedius* isolates, all isolates were methicillin susceptible (MSSP) (including two ST1115) (Table 3).

Clonally related *S. aureus* or *S. pseudintermedius* isolates were found in humans or dogs among 11.1% of households (*n* = 3). Two of the 16 households (household N^o^11 and 21) positive for nasal *S. aureus* had human carriers with similar clonal complexes (CCs), *spa-*types and IEC types (Table 2). In one of these households (N^o^ 11), MSSA-CC30-*spa*-type t1070 isolates (*scn*-negative) were identified in two humans, however, a different lineage, MSSA-CC8 of the *spa*-type t121 (IEC type-D), was identified in their dog (Table 4). In the second household (N° 21), two humans carried MSSA-CC398 isolates of different *spa*-types (t1451 and t571), although the dog was not *S. aureus* carrier. Moreover, in another household (N^o^ 10), a dog and a human were carriers of the same genetic lineage of *S. pseudintermedius* (MSSP-ST1115); in this household, the human also carried MSSA-CC97-t267 and a dog MSSA-t2013-CC15 (Table 4). All the *S. aureus* isolates were negative for *lukS/F*-PV*, tst-1, eta* and *etb* genes (Table 2). However, all the *S. pseudintermedius* isolates were positive for *lukS/F-I, siet,* and *sient* virulence genes, but one was only *sient*-positive (Tables 2, 3).

**Table 4:** Genetic lineages, virulence and AMR genes of CoPS isolates among households with both dog and human carriers.

Household ID N^o^/population	*S. aureus*						
	Isolate ID code/ host number	AMR	AMR genes	Virulence genes	IEC type	Plasmid replicons	*spa*-types/ST/CC
11/2 humans and 2 dogs	X6019/human 26	PEN	*blaZ*	*aur, cap8A-J, clfA, clfB, coa, cbp, fnbA, fnbB, hlB, hlD, icaA-D, icaR, isdA-G, hlgA, hlgB, hlgC, lukF-PV, vWbp, seg, sei, sem, sen, seo, seu*	Negative	rep5, rep16, rep19	t1070/ST30/CC30
	X6040/human 27	PEN	*blaZ*	*aur, cap8A-J, clfA, clfB, coa, cbp, fnbA, fnbB, hlB, hlD, icaA-D, icaR, isdA-G, hlgA, hlgB, hlgC, lukF-PV, vWbp, seg, sei, sem, sen, seo, seu*	Negative	rep5, rep16, rep19	t1070/ST30/CC30
	X6036/dog 29	PEN-CIP	*blaZ, grlA* (S80F)	*aur, cap8A-G, cap8L-P, clfA, clfB, coa, cbp, fnbA, fnbB, hlB, hlD, icaA-D, icaR, isdA-G, hlgA, hlgB, hlgC, lukF-PV, vWbp*	D	rep7, rep20	t121/ST8/CC8
10/2 humans and 2 dogs	X6061/human 23	Susceptible	NT	*aur, splA, splB, splE, hlgA, hlgB, hlgC, lukD-PV, lukE-PV*	E	None	t267/ST97/CC97
	X6065/Dog 24	PEN	*blaZ*	*aur, cap8A-P, clfA, clfB, coa, hlB, hlD, icaA-D, icaR, isdA-G, hlgA, hlgB, hlgC, lukF-PV, vWbp*	C	rep5, rep16	t2013/ST15/CC15
21/2 humans and 1 dogs	X6352/human 57	PEN-ERY-CLI^Ind^-GEN-TOB	*blaZ, ermT, aac6′-aph2″*	*aur, cap8A-G, cap8L-P, clfA, clfB, coa, cbp, fnbA, hlB, hlD, icaA-D, icaR, isdA-G, hlgA, hlgB, hlgC, vWbp*	C	rep13	t1451/ST398/CC398
	X6355/human 57	PEN-ERY-CLI^Ind^-GEN-TOB	*blaZ, ermT, aac6′-aph2″*	*aur, cap8A-G, cap8L-P, clfA, clfB, coa, cbp, fnbA, hlB, hlD, icaA-D, icaR, isdA-G, hlgA, hlgB, hlgC, vWbp*	C	rep13	t571/ST398/CC398
	X6353/human 58	PEN-ERY-CLI^Ind^-GEN-TOB	*blaZ, ermT, aac6′-aph2″*	*aur, cap8A-G, cap8L-P, clfA, clfB, coa, cbp, fnbA, hlB, hlD, icaA-D, icaR, isdA-G, hlgA, hlgB, hlgC, vWbp*	C	rep13	t1451/ST398/CC398
	X6358/human 58	PEN-ERY-CLI^Ind^-GEN-TOB	*blaZ, ermT, aac6′-aph2″*	*aur, cap8A-G, cap8L-P, clfA, clfB, coa, cbp, fnbA, hlB, hlD, icaA-D, icaR, isdA-G, hlgA, hlgB, hlgC, vWbp*	C	rep13	t571/ST398/CC398
Household ID N^o^/population	*S. pseudintermedius*						
	Isolate number/host	AMR	AMR genes	Virulence genes	IEC type	Plasmid replicons	ST
10/2 humans and 2 dogs	X6050/dog 25	Susceptible	NT	*lukS/F-I, siet, sient, clpP, hlgB*	Negative	rep7	ST1115
	X6059/human 23	Susceptible	NT	*lukS/F-I, siet, sient, clpP, hlgB*	Negative	rep7	ST1115

CIP, ciprofloxacin; ERY-CLI^ind^, erythromycin-clindamycin inducible; GEN, gentamicin; PEN, penicillin; TOB, tobramycin; NT, Not tested.

### Intra-host variation of genetic lineages or AMR genotypes of CoPS

Nine of the 16 *S. aureus* (56.3%) carriers harboured diverse *spa*-types or AMR genotypes in the same individual (dog or human). Of these, two to four genetically distinct *S. aureus* isolates were detected in these hosts (Table 2). In one human (ID number 3) with both MSSA and MRSA-SCC*mec* type-IV (2B) nasal carriage, three different MRSA-CC5-t2220 isolates with different AMR phenotypes/genes were detected: PEN-FOX-ERY-CLI-CIP-TOB-MUP-LZD/*blaZ, mecA, lnuA, msrA, mphC, mupA,* G2261A point mutation in 23S rDNA; PEN-FOX-ERY-CIP-GEN-TOB/*blaZ, mecA, aac6′-aph2″, msrA, mphC*; and PEN-FOX-ERY-CIP/*blaZ, mecA, msrA, mphC*, respectively; moreover, the MSSA isolate was typed as CC30-t012 and showed resistance only to PEN (*blaZ* positive) (Table 2). Two other humans (ID numbers 57 and 58) from the same household (N^o^ 21) carried *S. aureus* isolates both with similar genetic lineage (CC398) but different *spa* types (t571 and t1451) and similar AMR phenotypes (PEN-ERY-CLI^ind^-GEN-TOB) (Table 2). In another human *S. aureus* carrier (ID number 60) from a different household (N^o^ 22), isolates with different genetic lineages (CC15 and CC152) were detected (Table 2).

In the *S. pseudintermedius* isolates, 6 of the 12 carriers showed differences and intra-host variations in the AMR phenotypes or AMR genotypes. For instance, one of the dogs (ID number 52) harboured two different MSSP isolates (PEN-SXT/*blaZ, dfrA, dfrG* and PEN-SXT-TOB/*blaZ, dfrG, ant4′*) (Table 3). About 31.7% of the *S. pseudintermedius* had a MDR phenotype (Figure 2). All the three MRSA isolates and some MSSA isolates (20.0%) presented a MDR profile (Tables 2).

### Clonal relatedness of CoPS isolates within the same household

**Figure 3:**
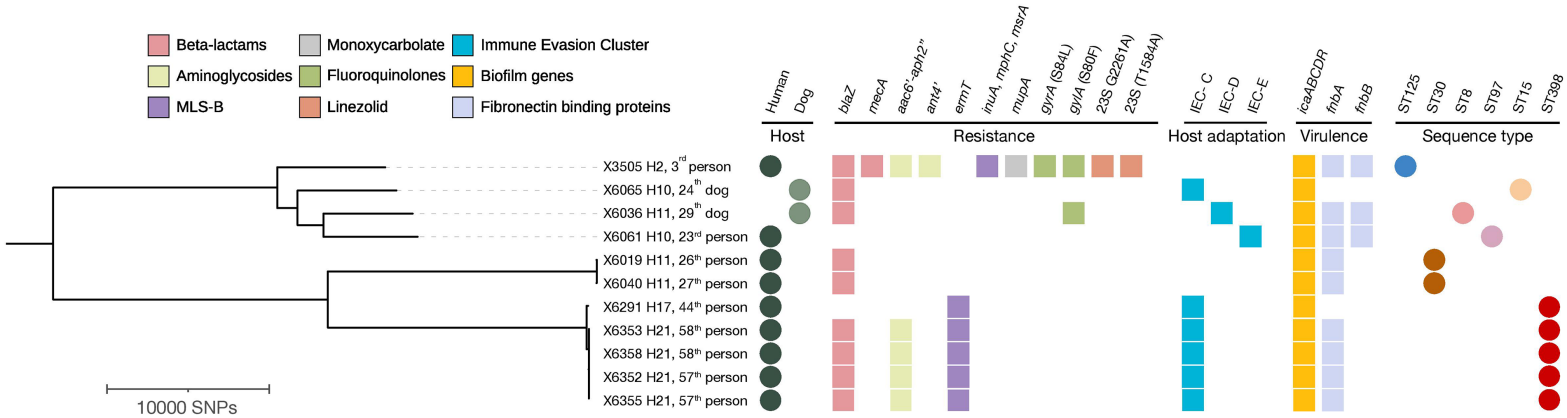
Phylogenetic tree based on core genome SNP analysis of 11 MRSA, MSSA-CC398, and other MSSA isolates from households with ≥2 humans and/or dogs’ carriers. The presence of antimicrobial resistance genes, immune evasion cluster (IEC), *icaABCDR* operon, fibronectin binding proteins, host type and sequence types (ST) are indicated. The filled circles indicate hosts and sequence types while the filled and squares indicate the confirmed antimicrobial resistance, host adaptation markers and virulence determinants extracted from MLST 2.0, ResFinder, PointFinder and VirulenceFinder. Visuals were obtained using iTOL v6.6. MLS-B, Macrolide-Lincosamide-Streptogramin-B. H, Household.

Upon WGS, very few SNPs difference (<15) were detected among *S. aureus* isolates from human carriers within the same household (N^o^ 10 and 21) (Figure 3), and these all shared the same repertoire of AMR genes, IEC types and virulence genes and plasmid replicons (Table 4; Figure 3). Concerning the MSSA-CC398 isolates, one isolate from a single person without a co-carrier in their household (N^o^ 17) had >250 SNP differences with those from another household (N^o^ 21) with two human MSSA-CC398 carriers (Supplementary Table S3). The major difference between these MSSA-CC398 isolates was the absence of the *blaZ, aac6′-aph2″,* and *fnbA* genes in the isolate from household number 17 (Figure 3).

Concerning the two *S. pseudintermedius*, each from a dog and human from the same household (N^o^ 10), they were both ST1115 NS identical (zero SNP differences) and shared identical virulence genes (Table 4).

## Discussion

Several studies have reported the transmission of CoPS between pets and their owners (Gómez-Sanz et al., 2013c; Han et al., 2016; Sahin-Tóth et al., 2021; Cuny et al., 2022). However, the present study is among the few that studied intra-species and within-host genetic diversities of CoPS in these hosts. Such information can better illustrate the complexity of challenges in the control of AMR in healthy dog-owning households.

The potential influence of dog-ownership on the nasal staphylococcal community (especially *S. aureus* and *S. pseudintermedius* colonization) of dogs and humans needs continuous surveillance. The present *S. aureus* household carriage rate (at least one dog and/or one human) in our study (44.4%) is lower than the 51.2% detected in a previous study performed a decade ago in Spain (Gómez-Sanz et al., 2013c), and the *S. aureus* carriage rate in humans in our study was also lower (34.1% *vs* 41.8%) (Gómez-Sanz et al., 2013c). Concerning studies from other European countries, our *S. aureus* prevalence in humans was higher than those reported in Germany (22%) and Hungary (23.8%) (Holtfreter et al., 2016; Sahin-Tóth et al., 2021).

Data on *S. aureus* nasal carriage rate among healthy dogs in community settings are sparsely available, varying between 2–8% according to different sources (Fazakerley et al., 2010; Rubin and Chirino-Trejo, 2010; Walther et al., 2012; Sahin-Tóth et al., 2021). Our results further support these numbers: we found a 5.9% carriage rate in dogs. However, in some African and Asian countries, high *S. aureus* nasal carriage is reported in healthy dogs, such as in Nigeria (36.9%), Indonesia (48.0%), Bangladesh (25.0%) and India (35.0%) (Mustapha et al., 2016; Sekhar et al., 2017; Rahman et al., 2018; Decline et al., 2020). The wide variation in nasal *S. aureus* carriage in dogs across the continents could be influenced by the local epidemiology of the *S. aureus*, differences in methodologies, dogs’ hygiene, environmental sanitation, antibiotic use in animals, and/or the health status of owners (Collignon and Voss, 2015; Fletcher, 2015; Valiakos et al., 2020).

As it was observed in this study, human *S. aureus* carriers were much more prevalent than dog carriers and indicating that transmission between humans and dogs occurs less frequently when compared to possible intrahousehold human-to-human transmission. Taking into consideration the molecular typing results, the genotypic profiles of *S. aureus* between our studied dog and human isolates were not similar, but those originating from humans in the same households were closely related in several cases, strongly suggesting human-to-human transmission. Moreover, there was a significant association between the household densities and nasal carriage of *S. pseudintermedius* in households with > than 1 dog and >than 1 human, pointing out for the possibility that a relatively higher household population is related to a higher detection rate of *S. pseudintermedius.*

The diverse pattern of the CCs of the MSSA isolates from dogs and in-contact humans corroborated the findings from a previous study in Germany and Spain (Gómez-Sanz et al., 2013c; Holtfreter et al., 2016). Here, a few human *S. aureus* isolates were *scn*-negative which indicates animal-adapted (perhaps dog-adapted) CCs of *S. aureus*. Nevertheless, dogs were carriers of MSSA-*scn*-positive isolates (CC8-IEC-type G and CC15-IEC-type C) suggesting an anthroponotic route of transmission. This is consistent with findings from a previous longitudinal study on *S. aureus* colonization dynamics in humans and dogs in Spain (Gómez-Sanz et al., 2013b). When compared to the current study, this suggests that humans are the major reservoir of *S. aureus* contributing to transmission to dogs. However, it is important to mention that zoonotic transmission from dogs to humans is also possible (Abdullahi et al., 2022).

Concerning *S. pseudintermedius*, 2.4 and 32.4% nasal carriage rates were detected in our studied humans and dogs, respectively. The *S. pseudintermedius* carriage rate among dogs in this study was higher than previously reported for dogs from Spain (22.7%) (Gómez-Sanz et al., 2013c), but lower than those reported in Canada (46%), Germany (37.5%), Australia (85.4%), Korea (65.9%), or Hungary (34.3%) (Hanselman et al., 2009; Bean and Wigmore, 2016; Han et al., 2016; Sahin-Tóth et al., 2021; Cuny et al., 2022). This suggests that the transfer between nasal *S. pseudintermedius* among healthy dogs could depend on the number of dogs in the household. This corroborated the previous reports of diverse MSSP lineages from Germany and France (Haenni et al., 2020; Cuny et al., 2022). Moreover, we did find nasal carriage of *S. pseudintermedius* among a person living with dogs in one of the investigated households. However, this is lower than previously reported in Korea, Spain, and Canada (Hanselman et al., 2009; Gómez-Sanz et al., 2013c; Han et al., 2016). It appears that humans are not natural hosts for *S. pseudintermedius*, but adaptation to humans could occur. A recent large study has shown diversity between the *S. pseudintermedius* isolates of human and dog infections with similar pathogenicity islands and virulence gene-containing prophages (Phumthanakorn et al., 2021). The household in our study with both human and dog *S. pseudintermedius* carriers strongly suggested intrahousehold transmission, as the isolates had no SNP (zero) difference and were confirmed as clones by our genomic analyses. To our knowledge, this lineage (ST1115) has not been reported so far for MSSP from dogs.

In our study, no MRSP was detected among healthy dogs and similar results were obtained in an study in Sweden (Börjesson et al., 2015). However, other studies refer low rates of MRSP nasal carriage in dogs, as 0.9% in Germany, 4.5% in Canada and 2.6% in Norway (Hanselman et al., 2009; Kjellman et al., 2015; Cuny et al., 2022). Moreover, a pooled 4.6% MRSP was reported among healthy dogs in Spain (Gómez-Sanz et al., 2011); of the nine MRSP nasal carriers, one was from a household dog, while the remaining eight were from stray dogs (Gómez-Sanz et al., 2011). The absence of MRSP in our study (healthy animals and household members) and the previously low MRSP in healthy dog studies are remarkably different to the high prevalence in dogs receiving treatment in veterinary clinics in France (16.9%) (Haenni et al., 2014). MRSP seems to be associated with animal-hospital-lineages (Ruiz-Ripa et al., 2021b), whereas isolates that are susceptible or have low AMR levels may represent natural colonizers of dogs. However, among our 19 isolates, three were found to harbour *dfrK*, a gene that is rarely detected in *S. pseudintermedius*, which could be linked to the mobile genetic element *Tn*559 (Ruzauskas et al., 2016; Ruiz-Ripa et al., 2021b). Further characterization of our isolate employing long-read sequencing could help to elucidate this possibility.

The MSSA-CC398 was a predominant lineage in our study, although detected only in humans. Evolutionarily, there are two clades of the CC398 based on the acquisition of SCC*mec* mobile element (carrying *mecA*), *Tn*916 (carrying *tet*(M)), or prophage φsa3 (carrying IEC), *viz:* (a) MRSA-CC398, often considered the predominant LA-MRSA clade (mostly IEC-negative) in Europe, (b) livestock-independent (human-adapted) clade of the MSSA-CC398 (often IEC-positive) (Price et al., 2012; Matuszewska et al., 2022). However, on very rare occasions, MRSA-CC398 carrying the φsa3 (*scn*-positive) and MSSA-CC398-*scn*-negative have been reported among certain *spa* types within the CC398 lineage (Price et al., 2012). Hence, these phenomena make it difficult to categorically classify the CC398 lineages into animal-adapted or human-adapted clades. However, it has been established that the CC398 lineage originated in MSSA from humans, acquired the tetracycline (*tet*(M)) and methicillin (*mecA*) genes and spread to livestock (Price et al., 2012). Thus, the absence of *tet*(M) in all the *S. aureus* and the detection of MSSA-CC398 clade could be inferred as humans-adapted strains. In many cases, MSSA-CC398 clade is associated with the predominant *spa* type t571 and the macrolide resistance gene *ermT* (Mama et al., 2021b). Worryingly, this MSSA-CC398 human clade has been recently considered an emergent lineage in invasive human infections in Spain and other countries (Laumay et al., 2021; Mama et al., 2021b). Concerning MSSA-CC398 in dog-owning households, a previous study by Gómez-Sanz et al. (2013c) also reported MSSA-CC398 of the *spa* type t571. An important similarity between the previous study and the current is that here, MSSA-CC398 isolates were only detected in humans and all were IEC type C of the *spa* type t571 and t1451. Recently, increased detection of penicillin susceptibility phenotype has been detected among invasive MSSA human isolates that have been causing clinical infections (Mama et al., 2021a). This phenotype has been frequently found among CC398 isolates (Mama et al., 2021a), however, most of our isolates are phenotypically resistant to penicillin. In another study by Gómez-Sanz et al. (2013a), about 7.1% of 98 kennel dogs also carried MSSA-CC398-*scn*-negative isolates but of different *spa* types (t034, t5883 and t108), and all were pan-susceptible. Our MSSA CC398 isolates were resistant at least to one antibiotic and worryingly 50% of the isolates were MDR. A major difference between the MSSA-CC398 isolates reported by Gómez-Sanz et al. (2013a) and ours was that here, human isolates were *scn*-positive (IEC type C). Recently in France, the MSSA-CC398 lineage (t571, t1451 and t18379) was also reported in 14.6 and 27.3% of dogs and cats, respectively (Tegegne et al., 2022). Worthy mentioning is the detection of MSSA-CC398-t571-*scn*-negative in a cat (Tegegne et al., 2022). The reason for this variation is not fully understood, however, it could be attributed to the *spa* type associated with the MSSA CC398 isolates or due to the carriage status of the Sa3 prophage (Gómez et al., 2020). The loss of Sa3int prophages in the *scn*-negative isolates is a major determinant for the human-to-animal transmission of MSSA CC398 (Price et al., 2012; Matuszewska et al., 2022). The findings of Gómez-Sanz et al. (2013a,c) and our study suggest the persistence of MSSA CC398 in humans and dogs.

Another finding of special epidemiological relevance is the dual MRSA/MSSA carriage detected in our study as both MSSA-CC30 and MRSA-CC5 were identified in a human household member. In a previous Spanish study, simultaneous carriage of both MRSA and MSSA of the CC398 lineage was reported in a farm worker with occupational exposure (Gómez et al., 2020). In another study among healthcare students in Portugal, concurrent detection of MRSA and MSSA in a single person was also reported (Coelho et al., 2021).

Though all the MRSA isolates had an MDR phenotype, more than 20% of the MSSA and MSSP isolates were also MDR. Generally, the AMR rate was moderate, but the most common AMR in *S. aureus* isolates were to penicillin, aminoglycosides, and erythromycin-clindamycin. Conversely, similarly to previous studies in *S. pseudintermedius*, the predominant AMR phenotype was to sulfamethoxazole-trimethoprim, erythromycin, and tetracycline (Rynhoud et al., 2021). Novel mutations (G2261A & T1584A) in the domain V region of the 23SrDNA of one MRSA isolate was observed, and although the predicted *in silico* resistance did not reveal a currently known AMR phenotype attributable to this mutation, the strain was phenotypically linezolid resistant. The inability to detect the linezolid phenotype from the genome database could be due to that this mutation has not been fully characterized (not previously reported and deposited in the genome database), as opposed to the most frequently detected 23S rDNA point mutation in staphylococci (G2576T) (Gostev et al., 2021; Ruiz-Ripa et al., 2021a). However, recently, novel point mutations in 23S rRNA associated with linezolid resistance in staphylococci have been reported in *S. epidermidis* in Austria (Huber et al., 2021) and in *S. capitis* in China (Han et al., 2022). It could be those novel mutations in domain V of 23S rRNA are silently emerging and mandates the need for close surveillance.

Regarding *S. aureus* virulence factors, all were negative for TSST-1, PVL, ETA and ETB encoding genes. All except one of the nasal *S. pseudintermedius* isolates of dogs and human origins carried the *lukS/F-I, siet,* and *expA* genes. These leucocidins and exfoliatins are responsible for host-specific clinical infections in dogs (Gharsa et al., 2013; Gómez-Sanz et al., 2013c).

Contrary to the findings of Penna et al. (2013), which reported *S. schleiferi* subsp. *schleiferi* carriage among ~32% of healthy dogs in Brazil, only 2 dogs from our study had *S. schleiferi* nasal carriage (5.4%). This finding is similar to the *S. coagulans*-positive dogs (4.9%) from the study of Lee et al. (2019) in Korea, but relatively higher than the 1.0% previously reported in Spain (Gómez-Sanz et al., 2013c). Since its first identification in humans in 1988 (Freney et al., 1988), several *S. coagulans* infections have been reported in humans and pets (May et al., 2005; Abraham et al., 2007; Yarbrough et al., 2017). Furthermore, the recent emergence of *S. coagulans* among healthy and pyodermic dogs has been a relevant global health issue in veterinary medicine due to its high AMR and diverse virulence factors (May et al., 2012; Abraham et al., 2007; Lee et al., 2019). Contrary to this assertion, all *S. coagulans* identified in our study were susceptible to antibiotics tested, likely because the isolates were cultured from healthy dogs.

It is worth mentioning a limitation of this study. As this was a one-point prospective study on the potential transmission of CoPS between dogs and dog-owner, randomly selected households were used. The sample size was relatively small and this could affect the detection of rare AMR phenotypes and virulence genes of CoPS in healthy individuals such as MRSP, *eta* and *tst.*

## Conclusion

The nasal carriage of *S. aureus* and *S. pseudintermedius* in healthy dogs’ households were moderate. Low rates of MRSA and *S. coagulans*, and no MRSP carriage were detected. Human-to-human MSSA and dog-to-human MSSP transmissions were identified in this study. *S. pseudintermedius* isolates had a homogeneous profile of virulence determinants. The detection of MSSA CC398, an emergent clade that has been implicated in invasive human infections is a relevant health concern and suggests the need for its continuous surveillance of humans, different species of animals and their shared environment from the One Health perspective.

## Data availability statement

The datasets presented in this study can be found in online repositories. The names of the repository/repositories and accession number(s) can be found in the article/Supplementary material.

## Ethics statement

The studies involving human and animal participants were reviewed and approved by Ethic Committe of the University of La Rioja, Spain. The patients/participants provided their written informed consent to participate in this study.

## Author contributions

IA and CT: conceptualization, methodology, and writing — original draft preparation. IA: laboratory experiments and software analysis. CT, IA, MZ, and CL: validation. IA, CT, MS, and AS: formal analysis and data curation. CT, IA, MS, AS, MZ, and CL: writing — review and editing. CT, CL, and MS: supervision. CT: project administration. CT, MZ, and IA: funding acquisition. All authors contributed to the article and approved the submitted version.

## Funding

This work was supported by the project PID2019-106158RB-I00 of the MCIN/AEI/10.13039/501100011033 of Spain. Also, it received funding from the European Union’s H2020 research and innovation programme under the Marie Sklodowska-Curie grant agreement N° 801586.

## Conflict of interest

The authors declare that the research was conducted in the absence of any commercial or financial relationships that could be construed as a potential conflict of interest.

## Publisher’s note

All claims expressed in this article are solely those of the authors and do not necessarily represent those of their affiliated organizations, or those of the publisher, the editors and the reviewers. Any product that may be evaluated in this article, or claim that may be made by its manufacturer, is not guaranteed or endorsed by the publisher.

## Footnotes

^1^ https://spa.ridom.de/submission.shtml

^2^ http://pubmlst.org/

^3^ https://itol.embl.de/

^4^ https://github.com/tseemann/mlst

^5^ https://github.com/tseemann/abricate

^6^ https://cge.cbs.dtu.dk/services

^7^ https://www.ebi.ac.uk/ena
