# Supplementary material for: Clonal relatedness of coagulase-positive staphylococci among healthy dogs and dog-owners in Spain. Detection of multidrug-resistant-MSSA-CC398 and novel linezolid-resistant-MRSA-CC5
Source: Front Microbiol. 2023 Mar 2;14:1121564. doi: 10.3389/fmicb.2023.1121564 (PMC10017961; doi:10.3389/fmicb.2023.1121564)
Supplement: Supplementary file 1 [file Table_1.DOCX]

| **Gene** | **Primers’ oligonucleotide (5’**⇒**3’)** | **Amplicon size** | **Reference** |
| --- | --- | --- | --- |
| **Antimicrobial resistance (AMR) genes** | | | |
| *blaZ* | F: CAGTTCACATGCCAAAGAG | 772 bp | (Schnellmann et al., 2006) |
|  | R: TACACTCTTGGCGGTTTC |  |  |
| *mecA* | F: GGGATCATAGCGTCATTATTC | 527 bp | (Poulsen et al., 2013) |
|  | R: AACGATTGTGACACGATAGCC |  |  |
| *mecC* | F: GCTCCTAATGCTAATGCA | 304 bp | (Cuny et al., 2011) |
|  | R: TAAGCAATAATGACTACC |  |  |
| *ermA* | F: TCTAAAAAGCATGTAAAAGAA | 645 bp | (Sutcliffe et al., 1996) |
|  | R: CTTCGATAGTTTATTAATATTAG |  |  |
| *ermB* | F: GAAAAGTACTCAACCAAATA | 639 bp | (Sutcliffe et al., 1996) |
|  | R: AGTAACGGTACTTAAATTGTTTA |  |  |
| *ermC* | F: TCAAAACATAATATAGATAAA | 642 bp | (Sutcliffe et al., 1996) |
|  | R: GCTAATATTGTTTAAATCGTCAAT |  |  |
| *ermT* | F: CCGCCATTGAAATAGATCCT | 200 bp | (Gómez-Sanz et al., 2010) |
|  | R: TTCTGTAGCTGTGCTTTCAAAAA |  |  |
| *mphC* | F: ATGACTCGACATAATGAAAT | 900 bp | (Schnellmann et al., 2006) |
|  | R: CTACTCTTTCATACCTAACTC |  |  |
| *msrA* | F: GCAAATGGTGTAGGTAAGACAACT | 399 bp | (Wondrack et al., 1996) |
|  | R: ATCATGTGATGTAAACAAAAT |  |  |
| *lnuA* | F: GGTGGCTGGGGGGTAGATGTATTAACTGG | 323 bp | (Lina et al., 1999) |
|  | R: GCTTCTTTTGAAATACATGGTATTTTTCGATC |  |  |
| *lnuB* | F: CCTACCTATTGTTTGTGGAA | 499 bp | (Bozdogan et al., 1999) |
|  | R: ATAACGTTACTCTCCTATTC |  |  |
| *aac6’-aph2’’* | F: CCAAGAGCAATAAGGGCATA | 220 bp | (Van de Klundert and Vliegenthart 1993) |
|  | R: CACTATCATAACCACTACCG |  |  |
| *ant4’* | F: GCAAGGACCGACAACATTTC | 165 bp | (Van de Klundert and Vliegenthart 1993) |
|  | R: TGGCACAGATGGTCATAACC |  |  |
| *tet*(L) | F: CATTTGGTCTTATTGGATCG | 456 bp | (Aarestrup et al., 2000) |
|  | R: ATTACACTTCCGATTTCGG |  |  |
| *tet*(M) | F: GTTAAATAGTGTTCTTGGAG | 576 bp | (Aarestrup et al., 2000) |
|  | R: CTAAGATATGGCTCTAACAA |  |  |
| *tet*(K) | F: TTAGGTGAAGGGTTAGGTCC | 697 bp | (Aarestrup et al., 2000) |
|  | R: GCAAACTCATTCCAGAAGCA |  |  |
| *dfrA* | F: CCTTGGCACTTACCAAATG | 374 bp | (Schnellmann et al., 2006) |
|  | R: CTGAAGATTCGACTTCCC |  |  |
| *dfrD* | F: TTCTTTAATTGTTGCGATGG | 582 bp | (Schnellmann et al., 2006) |
|  | R: TTAACGAATTCTCTCATATATATG |  |  |
| *dfrG* | F: TCGGAAGAGCCTTACCTGACAGAA | 323 bp | (Gómez-Sanz et al., 2010) |
|  | R: CCCTTTTTGGGCAAATACCTCATTCCA |  |  |
| *dfrK* | F: GAGAATCCCAGAGGATTGGG | 423 bp | (Gómez-Sanz et al., 2010) |
|  | R: CAAGAAGCTTTTCGCTCATAAA |  |  |
| *cat_pC221_* | F: ATTTATGCAATTATGGAAGTTG | 434 bp | (Schnellmann et al., 2006) |
|  | R: TGAAGCATGGTAACCATCAC |  |  |
| *cat_pC223_* | F: GAATCAAATGCTAGTTTTAACTC | 283 bp | (Schnellmann et al., 2006) |
|  | R: ACATGGTAACCATCACATAC |  |  |
| *cat_pC194_* | F: CGACTTTTAGTATAACCACAGA | 570 bp | (Schnellmann et al., 2006) |
|  | R: GCCAGTCATTAGGCCTAT |  |  |
| *catA* | F: GGATATGAAATTTATCCCTC | 505 bp | (Aarestrup et al., 2000) |
|  | R: CAATCATCTACCCTATGAAT |  |  |
| *fexA* | F: GTACTTGTAGGTGCAATTACGGCTGA | 1272 bp | (Kehrenberg et al., 2005) |
|  | R: CGCATCTGAGTAGGACATAGCGTC |  |  |
| *fexB* | F: TTCCCACTATTGGTGAAAGGAT | 816 bp | (Liu et al., 2012) |
|  | R: GCAATTCCCTTTTATGGACGTT |  |  |
| *cfr* | F: TGAAGTATAAAGCAGGTTGGGAGTCA | 746 bp | (Kehrenberg et al., 2006) |
|  | R: ACCATATAATTGACCACAAGCAGC |  |  |
| *cfrB* | F: TGAGCATATACGAGTAACCTCAAGA | 293 bp | (Lee et al., 2017) |
|  | R: CGCAAGCAGCGTCTATATCA |  |  |
| *cfrD* | F: AGAAGTCGCAACAAGTGAGGA | 595 bp | (Ruiz-Ripa et al., 2020) |
|  | R: GCAACTGCATGAGTCAAAGAA |  |  |
| *optrA* | F: AGGTGGTCAGCGAACTAA | 1395 bp | (Wang et al., 2015) |
|  | R: ATCAACTGTTCCCATTCA |  |  |
| *poxtA* | F: TCAATGCAGAGCAGGAAGCA | 791 bp | (Ruiz-Ripa et al., 2020) |
|  | R: GGTGGATTTACCGACACCGT |  |  |
| *23S-rDNA* | F: GCGGTCGCCTCCTAAAAG | 420 bp | (Dibo et al., 2004) |
|  | R: ATCCCGGTCCTCTCGTACT |  |  |
| *mupA* | F: CCCATGGCTTACCAGTTGA | 419 pb | (Udo et al., 2003) |
|  | R: CCATGGAGCACTATCCGAA |  |  |
| **IEC and virulence genes** | | | |
| *scn* | F: AGCACAAGCTTGCCAACATCG | 257 bp | (Van Wamel et al., 2006) |
|  | R: TTAATATTTACTTTTTAGTGC |  |  |
| *chp* | F: TTTACTTTTGAACCGTTTCCTAC | 366 bp | (Van Wamel et al., 2006) |
|  | R: CGTCCTGAATTCTTAGTATGCATATTCATTAG |  |  |
| *sak* | F: AAGGCGATGACGCGAGTTAT | 223 bp | (Van Wamel et al., 2006) |
|  | R: GCGCTTGGATCTAATTCAAC |  |  |
| *sea* | F: AGATCATTCGTGGTATAACG | 344 bp | (Van Wamel et al., 2006) |
|  | R: TTAACCGAAGGTTCTGTAGA |  |  |
| *sep* | F: AATCATAACCAACCGAATCA | 196 bp | (Van Wamel et al., 2006) |
|  | R: TCATAATGGAAGTGCTATAA |  |  |
| *lukF-I* | F: CCTGTCTATGCCGCTAATCAA | 572 bp | (Futagawa-Saito Yamaguchi et al., 2002) |
|  | R: AGGTCATGGAAGCTATCTCGA |  |  |
| *lukS-I* | F: TGTAAGCAGCAGAAAATGGGG | 332 bp | (Futagawa-Saito Yamaguchi et al., 2002) |
|  | R: GCCCGATAGGACTTCTTACAA |  |  |
| *siet* | F: ATGGAAAATTTAGCGGCATCTGG | 359 bp | (Lautz et al., 2006) |
|  | R: CCATTACTTTTCGCTTGTTGTGC |  |  |
| *sient* | F: GCAAGCATATCATTACATTTG | 147 bp | (Futagawa-Saito Yamaguchi et al., 2002) |
|  | R: ACTTGATATACCCTGTTTCGT |  |  |
| *expB* | F: GGGCATGCACATATGATGAAGCC | 740 bp | (Iyori et al., 2010) |
|  | R: CCAGATCTATCTTCTGATTCAGC |  |  |
| *tst* | F: TTCACTATTTGTAAAAGTGTCAGACCCACT | 180 bp | (Yamaguchi et al., 2002) |
|  | R: TACTAATGAATTTTTTTATCGTAAGCCCTT |  |  |
| *lukS/F*-PV | F: ATCATTAGGTAAAATGTCTGGACATGATCCA | 443 bp | (Lina et al., 1999) |
|  | R: GCATCAAGTGTATTGGATAGCAAAAGC |  |  |
| *eta* | F: ACTGTAGGAGCTAGTGCATTTGT | 190 bp | (Jarraud et al., 2002) |
|  | R: TGGATACTTTTGTCTATCTTTTTCATCAAC |  |  |
| *etb* | F: CAGATAAAGAGCTTTATACACACATTAC | 612 bp | (Jarraud et al., 2002) |
|  | R: AGTGAACTTATCTTTCTATTGAAAAACACTC |  |  |
| ***S. pseudintermedius* housekeeping alleles for MLST** | | | |
| *pta* | F: GTGCGTATCGTATTACCAGAAGG | 570 pb | (Bannoehr et al., 2007) |
|  | R: GCAGAACCTTTTGTTGAGAAGC |  |  |
| *cpn60* | F: GCGACTGTACTTGCACAAGCA | 552 pb | (Bannoehr et al., 2007) |
|  | R: AACTGCAACCGCTGTAAATG |  |  |
| *tuf* | F: CAATGCCACAAACTCG | 500 pb | (Bannoehr et al., 2007) |
|  | R: GCTTCAGCGTAGTCTA |  |  |
| *ack* | F: CACCACTTCACAACCCAGCAAACT | 680 bp | (Solyman et al., 2013) |
|  | R: AACCTTCTAATACACGCGCACGCA |  |  |
| *purA* | F: GATTACTTCCAAGGTATGTTT | 490 bp | (Solyman et al., 2013) |
|  | R: TCGATAGAGTTAATAGATAAGTC |  |  |
| *Sar* | F: GGATTTAGTCCAGTTCAAAATTT | 521 bp | (Solyman et al., 2013) |
|  | R: GAACCATTCGCCCCATGAA |  |  |
| *fdh* | F: TGCGATAACAGGATGTGCTT | 408 bp | (Solyman et al., 2013) |
|  | R: CTTCTCATGATTCACCGGC |  |  |
| **Staphylococcal Protein A typing** | | | |
| *spa* | F: AGACGATCCTTCGGTGAGC | Hypervariable | (Harmsen et al., 2003) |
|  | R: GCTTTTGCAATGTCATTTACTG |  |  |
| **SCC*mec* types** | | | |
| *SCCmecI* | F: GCTTTAAAGAGTGTCGTTACAGG | 613 bp | (Zhang et al., 2005) |
|  | R: GTTCTCTCATAGTATGACGTCC |  |  |
| *SCCmecII* | F: CGTTGAAGATGATGAAGCG | 398 bp | (Zhang et al., 2005) |
|  | R: CGAAATCAATGGTTAATGGACC |  |  |
| *SCCmecIII* | F: CCATATTGTGTACGATGCG | 280 bp | (Zhang et al., 2005) |
|  | R: CCTTAGTTGTCGTAACAGATCG |  |  |
| *SCCmecIVa* | F: GCCTTATTCGAAGAAACCG | 776 bp | (Zhang et al., 2005) |
|  | R: CTACTCTTCTGAAAAGCGTCG |  |  |
| *SCCmecIVb* | F: TCTGGAATTACTTCAGCTGC | 493 bp | (Zhang et al., 2005) |
|  | R: AAACAATATTGCTCTCCCTC |  |  |
| *SCCmecIVc* | F: ACAATATTTGTATTATCGGAGAGC | 200 bp | (Zhang et al., 2005) |
|  | R: TTGGTATGAGGTATTGCTGG |  |  |
| *SCCmecIVd* | F: CTCAAAATACGGACCCCAATACA | 881 bp | (Zhang et al., 2005) |
|  | R: TGCTCCAGTAATTGCTAAAG |  |  |
| *SCCmecV* | F: GAACATTGTTACTTAAATGAGCG | 325 bp | (Zhang et al., 2005) |
|  | R: TGAAAGTTGTACCCTTGACACC |  |  |
| **CC398 lineage** | | | |
| *sau1-hsdS1* | F: AGGGTTTGAAGGCGAATGGG | 296 bp | (Stegger et al., 2011) |
|  | R: CAGTATAAAGAGGTGACATGACCCCT |  |  |

Aarestrup, F. M., Agerso, Y., Gerner-Smidt, P., Madsen, M., & Jensen, L. B. (2000). Comparison of antimicrobial resistance phenotypes and resistance genes in Enterococcus faecalis and Enterococcus faecium from humans in the community, broilers, and pigs in Denmark. *Diagnostic microbiology and infectious disease*, *37*(2), 127–137. <https://doi.org/10.1016/s0732-8893(00)00130-9>

Bannoehr, J., Ben Zakour, N. L., Waller, A. S., Guardabassi, L., Thoday, K. L., van den Broek, A. H., & Fitzgerald, J. R. (2007). Population genetic structure of the Staphylococcus intermedius group: insights into agr diversification and the emergence of methicillin-resistant strains. *Journal of bacteriology*, *189*(23), 8685–8692. <https://doi.org/10.1128/JB.01150-07>

Bozdogan, B., Berrezouga, L., Kuo, M. S., Yurek, D. A., Farley, K. A., Stockman, B. J., & Leclercq, R. (1999). A new resistance gene, linB, conferring resistance to lincosamides by nucleotidylation in Enterococcus faecium HM1025. *Antimicrobial agents and chemotherapy*, *43*(4), 925–929. <https://doi.org/10.1128/AAC.43.4.925>

Cuny, C., Layer, F., Strommenger, B., & Witte, W. (2011). Rare occurrence of methicillin-resistant Staphylococcus aureus CC130 with a novel mecA homologue in humans in Germany. *PloS one*, *6*(9), e24360. <https://doi.org/10.1371/journal.pone.0024360>

Dibo, I., Pillai, S. K., Gold, H. S., Baer, M. R., Wetzler, M., Slack, J. L., Hazamy, P. A., Ball, D., Hsiao, C. B., McCarthy, P. L., Jr, & Segal, B. H. (2004). Linezolid-resistant Enterococcus faecalis isolated from a cord blood transplant recipient. *Journal of clinical microbiology*, *42*(4), 1843–1845. <https://doi.org/10.1128/JCM.42.4.1843-1845.2004>

Dibo, I., Pillai, S. K., Gold, H. S., Baer, M. R., Wetzler, M., Slack, J. L., Hazamy, P. A., Ball, D., Hsiao, C. B., McCarthy, P. L., Jr, & Segal, B. H. (2004). Linezolid-resistant Enterococcus faecalis isolated from a cord blood transplant recipient. *Journal of clinical microbiology*, *42*(4), 1843–1845. <https://doi.org/10.1128/JCM.42.4.1843-1845.2004>

Gómez-Sanz, E., Torres, C., Lozano, C., Fernández-Pérez, R., Aspiroz, C., Ruiz-Larrea, F., & Zarazaga, M. (2010). Detection, molecular characterization, and clonal diversity of methicillin-resistant Staphylococcus aureus CC398 and CC97 in Spanish slaughter pigs of different age groups. *Foodborne pathogens and disease*, *7*(10), 1269–1277. <https://doi.org/10.1089/fpd.2010.0610>

Harmsen, D., Claus, H., Witte, W., Rothgänger, J., Claus, H., Turnwald, D., & Vogel, U. (2003). Typing of methicillin-resistant Staphylococcus aureus in a university hospital setting by using novel software for spa repeat determination and database management. *Journal of clinical microbiology*, *41*(12), 5442–5448. <https://doi.org/10.1128/JCM.41.12.5442-5448.2003>

Iyori, K., Hisatsune, J., Kawakami, T., Shibata, S., Murayama, N., Ide, K., Nagata, M., Fukata, T., Iwasaki, T., Oshima, K., Hattori, M., Sugai, M., & Nishifuji, K. (2010). Identification of a novel Staphylococcus pseudintermedius exfoliative toxin gene and its prevalence in isolates from canines with pyoderma and healthy dogs. *FEMS microbiology letters*, *312*(2), 169–175. <https://doi.org/10.1111/j.1574-6968.2010.02113.x>

Jarraud, S., Mougel, C., Thioulouse, J., Lina, G., Meugnier, H., Forey, F., Nesme, X., Etienne, J., & Vandenesch, F. (2002). Relationships between Staphylococcus aureus genetic background, virulence factors, agr groups (alleles), and human disease. *Infection and immunity*, *70*(2), 631–641. <https://doi.org/10.1128/IAI.70.2.631-641.2002>

Kehrenberg, C., & Schwarz, S. (2006). Distribution of florfenicol resistance genes fexA and cfr among chloramphenicol-resistant Staphylococcus isolates. *Antimicrobial agents and chemotherapy*, *50*(4), 1156–1163. <https://doi.org/10.1128/AAC.50.4.1156-1163.2006>

Kehrenberg, C., & Schwarz, S. (2005). Florfenicol-chloramphenicol exporter gene fexA is part of the novel transposon Tn558. *Antimicrobial agents and chemotherapy*, *49*(2), 813–815. <https://doi.org/10.1128/AAC.49.2.813-815.2005>

Lautz, S., Kanbar, T., Alber, J., Lämmler, C., Weiss, R., Prenger-Berninghoff, E., & Zschöck, M. (2006). Dissemination of the gene encoding exfoliative toxin of Staphylococcus intermedius among strains isolated from dogs during routine microbiological diagnostics. *Journal of veterinary medicine. B, Infectious diseases and veterinary public health*, *53*(9), 434–438. <https://doi.org/10.1111/j.1439-0450.2006.00999.x>

Lee, S. M., Huh, H. J., Song, D. J., Shim, H. J., Park, K. S., Kang, C. I., Ki, C. S., & Lee, N. Y. (2017). Resistance mechanisms of linezolid-nonsusceptible enterococci in Korea: low rate of 23S rRNA mutations in Enterococcus faecium. *Journal of medical microbiology*, *66*(12), 1730–1735. <https://doi.org/10.1099/jmm.0.000637>

Lina, G., Quaglia, A., Reverdy, M. E., Leclercq, R., Vandenesch, F., & Etienne, J. (1999). Distribution of genes encoding resistance to macrolides, lincosamides, and streptogramins among staphylococci. *Antimicrobial agents and chemotherapy*, *43*(5), 1062–1066. <https://doi.org/10.1128/AAC.43.5.1062>

Liu, H., Wang, Y., Wu, C., Schwarz, S., Shen, Z., Jeon, B., Ding, S., Zhang, Q., & Shen, J. (2012). A novel phenicol exporter gene, fexB, found in enterococci of animal origin. *The Journal of antimicrobial chemotherapy*, *67*(2), 322–325. <https://doi.org/10.1093/jac/dkr481>

Poulsen, A. B., Skov, R., & Pallesen, L. V. (2003). Detection of methicillin resistance in coagulase-negative staphylococci and in staphylococci directly from simulated blood cultures using the EVIGENE MRSA Detection Kit. *The Journal of antimicrobial chemotherapy*, *51*(2), 419–421. <https://doi.org/10.1093/jac/dkg084>

Ruiz-Ripa, L., Feßler, A. T., Hanke, D., Eichhorn, I., Azcona-Gutiérrez, J. M., Pérez-Moreno, M. O., Seral, C., Aspiroz, C., Alonso, C. A., Torres, L., Alós, J. I., Schwarz, S., & Torres, C. (2020). Mechanisms of Linezolid Resistance Among Enterococci of Clinical Origin in Spain-Detection of *optrA*- and *cfr*(D)-Carrying *E.faecalis*. *Microorganisms*, *8*(8), 1155. <https://doi.org/10.3390/microorganisms8081155>

Schnellmann, C., Gerber, V., Rossano, A., Jaquier, V., Panchaud, Y., Doherr, M. G., Thomann, A., Straub, R., & Perreten, V. (2006). Presence of new mecA and mph(C) variants conferring antibiotic resistance in Staphylococcus spp. isolated from the skin of horses before and after clinic admission. *Journal of clinical microbiology*, *44*(12), 4444–4454. <https://doi.org/10.1128/JCM.00868-06>

Solyman, S. M., Black, C. C., Duim, B., Perreten, V., van Duijkeren, E., Wagenaar, J. A., Eberlein, L. C., Sadeghi, L. N., Videla, R., Bemis, D. A., & Kania, S. A. (2013). Multilocus sequence typing for characterization of Staphylococcus pseudintermedius. *Journal of clinical microbiology*, *51*(1), 306–310. <https://doi.org/10.1128/JCM.02421-12>

Stegger, M., Lindsay, J. A., Moodley, A., Skov, R., Broens, E. M., & Guardabassi, L. (2011). Rapid PCR detection of Staphylococcus aureus clonal complex 398 by targeting the restriction-modification system carrying sau1-hsdS1. *Journal of clinical microbiology*, *49*(2), 732–734. <https://doi.org/10.1128/JCM.01970-10>

Sutcliffe, J., Grebe, T., Tait-Kamradt, A., & Wondrack, L. (1996). Detection of erythromycin-resistant determinants by PCR. *Antimicrobial agents and chemotherapy*, *40*(11), 2562–2566. <https://doi.org/10.1128/AAC.40.11.2562>

Udo, E. E., Al-Sweih, N., & Noronha, B. C. (2003). A chromosomal location of the mupA gene in Staphylococcus aureus expressing high-level mupirocin resistance. *The Journal of antimicrobial chemotherapy*, *51*(5), 1283–1286. <https://doi.org/10.1093/jac/dkg188>

Van de Klundert, J., Vliegenthart, J. (1993). PCR detection of genes coding for aminoglycoside-modifying enzymes, in: Diagnostic Molecular Microbiology. Principles and Applications. pp. 547– 552. <https://doi.org/10.1023/A:1016601629518>

van Wamel, W. J., Rooijakkers, S. H., Ruyken, M., van Kessel, K. P., & van Strijp, J. A. (2006). The innate immune modulators staphylococcal complement inhibitor and chemotaxis inhibitory protein of Staphylococcus aureus are located on beta-hemolysin-converting bacteriophages. *Journal of bacteriology*, *188*(4), 1310–1315. <https://doi.org/10.1128/JB.188.4.1310-1315.2006>

Wang, Y., Lv, Y., Cai, J., Schwarz, S., Cui, L., Hu, Z., Zhang, R., Li, J., Zhao, Q., He, T., Wang, D., Wang, Z., Shen, Y., Li, Y., Feßler, A. T., Wu, C., Yu, H., Deng, X., Xia, X., & Shen, J. (2015). A novel gene, optrA, that confers transferable resistance to oxazolidinones and phenicols and its presence in Enterococcus faecalis and Enterococcus faecium of human and animal origin. *The Journal of antimicrobial chemotherapy*, *70*(8), 2182–2190. <https://doi.org/10.1093/jac/dkv116>

Wondrack, L., Massa, M., Yang, B. V., & Sutcliffe, J. (1996). Clinical strain of Staphylococcus aureus inactivates and causes efflux of macrolides. *Antimicrobial agents and chemotherapy*, *40*(4), 992–998. <https://doi.org/10.1128/AAC.40.4.992>

Yamaguchi, T., Nishifuji, K., Sasaki, M., Fudaba, Y., Aepfelbacher, M., Takata, T., Ohara, M., Komatsuzawa, H., Amagai, M., & Sugai, M. (2002). Identification of the Staphylococcus aureus etd pathogenicity island which encodes a novel exfoliative toxin, ETD, and EDIN-B. *Infection and immunity*, *70*(10), 5835–5845. <https://doi.org/10.1128/IAI.70.10.5835-5845.2002>

Zhang, K., McClure, J. A., Elsayed, S., Louie, T., & Conly, J. M. (2005). Novel multiplex PCR assay for characterization and concomitant subtyping of staphylococcal cassette chromosome mec types I to V in methicillin-resistant Staphylococcus aureus. *Journal of clinical microbiology*, *43*(10), 5026–5033. <https://doi.org/10.1128/JCM.43.10.5026-5033.2005>
