# Supplementary material for: Clonal relatedness of coagulase-positive staphylococci among healthy dogs and dog-owners in Spain. Detection of multidrug-resistant-MSSA-CC398 and novel linezolid-resistant-MRSA-CC5
Source: Front Microbiol. 2023 Mar 2;14:1121564. doi: 10.3389/fmicb.2023.1121564 (PMC10017961; doi:10.3389/fmicb.2023.1121564)
Supplement: Supplementary file 2 [file Table_2.DOCX]

**Table S2.** Samples of humans and dogs tested, and species of coagulase-positive staphylococci detected. ___________________________________________________________________________________________________________________________________________

No. households No. of humans N^o^ of dogs N^o^ of households positive for: N^o^ of isolates from humans/dogs *N^o^ of distinct isolates from

of the species: humans/dogs of species^a^:

SA SP SC SA SP SC SA SP SC

27 41 24 16 12 1 73 31 2 31 19 2

SA: *S. aureus;* SP: *S. pseudintermedius;* SC*: S. coagulans*

^a^One isolate per sample or more than one when they were of different species and/or different AMR phenotype
